# Flexible Capacitive Pressure Sensors with Ultrasonically Engineered Cu-Filled PDMS Dielectric Layers

**DOI:** 10.3390/s26123721

**Published:** 2026-06-11

**Authors:** Xuelei Jia, Zhiwei Xu, Jiahao Huang, Yinlong Zhu, Shuang Xi, Junchao Zhang, Xu Wang

**Affiliations:** 1 College of Mechanical and Electronic Engineering, Nanjing Forestry University, Nanjing 210037, China; 2311501110@njfu.edu.cn (X.J.); xuzwei@njfu.edu.cn (Z.X.); huangjiahao@njfu.edu.cn (J.H.); ylzhu@njfu.edu.cn (Y.Z.); shuangxi@njfu.edu.cn (S.X.); 2 State Key Laboratory of Robotics, Shenyang Institute of Automation, Chinese Academy of Sciences, Shenyang 110169, China; 3 College of Aeronautics and Astronautics, Nanjing University of Aeronautics and Astronautics, Nanjing 210016, China; 15565369735@163.com

**Keywords:** flexible capacitive sensor, Cu/PDMS composite dielectric, ultrasonic processing, particle dispersion, dielectric modulation

## Abstract

Flexible capacitive pressure sensors have garnered significant attention in wearable electronics and robotic tactile sensing due to their high flexibility and simple structure. However, non-uniform distribution of conductive fillers in composite dielectric layers often compromises dielectric stability and sensing performance. In this work, a Cu/PDMS composite dielectric layer was fabricated using ultrasonic-assisted homogenization to enhance Cu particle dispersion and suppress sedimentation. A theoretical model and finite element simulations were employed to investigate the effects of particle distribution on permittivity, capacitance, electric field, and current density. The results indicate that uniform Cu dispersion improves dielectric stability and mitigates local electric-field concentration. Compared with conventionally prepared sensors, the ultrasonically treated sensor demonstrated higher sensitivity, enhanced dielectric stability, and a broader working range. Specifically, the sensor achieved a sensitivity of 0.157 kPa^−1^ within 0–1 kPa and maintained stable performance over 1000 loading cycles. These findings confirm that ultrasonic-assisted homogenization is an effective approach for improving the dielectric and sensing performance of flexible capacitive pressure sensors.

## 1. Introduction

With the rapid development of wearable electronics, flexible robotics, and intelligent healthcare, conventional rigid pressure sensors are increasingly inadequate for applications requiring conformal contact with curved surfaces and dynamic deformation [1,2,3]. Owing to their superior compliance and stretchability, flexible pressure sensors, particularly those based on conductive hydrogels with self-healing capability, have shown great potential in health monitoring and human–machine interaction [4,5,6,7]. Based on their sensing mechanisms, flexible pressure sensors can be classified into capacitive, resistive, piezoelectric, and triboelectric types [8,9,10,11]. Recently, emerging mechanisms such as LC resonant, magnetoelastic, and electrochemical sensing have also attracted increasing attention [12,13,14,15,16].

Among these, capacitive sensors are particularly attractive owing to their low power consumption, minimal hysteresis, high stability, and good dynamic response, and thus they are widely employed for physiological signal detection and tactile sensing [4,17,18,19]. Their performance depends on electrode area, inter-electrode distance, and the dielectric constant of the dielectric layer [20,21]. Recently, iontronic materials have emerged as a promising strategy to further overcome the limitations of conventional capacitive sensors, owing to their ultrahigh interfacial capacitance and excellent low-pressures sensitivity [22].

Sensitivity, linearity, and cyclic stability are key metrics for flexible pressure sensors. Sensitivity reflects the ability to detect pressure variations, with compressive strain of the dielectric layer being a primary factor enhancing it [23,24,25]. Nevertheless, capacitive sensors still face challenges, including high sensitivity at low pressures accompanied by rapid attenuation at higher pressures, narrow linear range, and trade-offs between mechanical and dielectric properties [22,26,27]. Fabrication techniques may also be limited by high cost, scalability issues, or insufficient precision [2,28,29].

Current strategies to overcome these limitations fall into two categories. The first involves microstructural design of the dielectric layer to enhance compressive deformation and improve low-pressure sensitivity [17,30]. For example, Lee et al. developed a capacitive sensor with an arched microstructure, achieving a sensitivity of 0.116 kPa^−1^ in the low-pressure range—about 30 times higher than an unstructured substrate—while maintaining a low detection limit and good cyclic stability [31]. The second approach incorporates conductive fillers into the dielectric matrix to increase the dielectric constant and enhance pressure response [32]. For instance, Chang et al. improved sensor sensitivity (~1.99 kPa^−1^) and response speed by tuning MWCNT content and surface structure [33]. However, filler agglomeration and increased dielectric loss can compromise device uniformity [34,35].

PDMS is widely regarded as an ideal flexible dielectric material due to its inherent flexibility, ease of processing, and tunable mechanical properties [36]. Micro-copper particles were selected as fillers due to their high electrical conductivity and cost-effectiveness, making them more suitable for large-scale fabrication. Incorporating Cu particles into PDMS can effectively enhance the dielectric constant and pressure response. However, the large density mismatch between Cu and PDMS often induces particle sedimentation during curing, leading to non-uniform distribution, localized electric-field concentration, and degraded device reliability. Existing mitigation strategies often compromise mechanical flexibility, increase fabrication cost, or lack scalability [37,38,39]. Acoustic-assisted particle manipulation has been shown to enable controllable concentration and transport of micro/nanoparticles, offering a potential strategy for improving filler dispersion uniformity in polymer matrices [40].

To overcome these challenges, we propose an ultrasonic vibration-assisted dispersion strategy to enhance the distribution of Cu particles with the PDMS matrix without introducing additional phases or fillers. By mitigating particle sedimentation during composite fabrication, flexible capacitive pressure sensors with improved dielectric uniformity and overall performance are achieved. The relationship between particle sedimentation and dielectric properties is investigated through theoretical modeling. Specifically, a multilayer series-capacitance model is established along the concentration-gradient direction, incorporating a concentration–permittivity relationship derived from percolation theory. In addition, finite element simulations are performed for both sedimented and uniformly dispersed particle configurations to elucidate the influence of particle distribution on dielectric behavior. The distributions of electric potential, electric field, and local current density are further analyzed near the percolation threshold.

The fabricated sensors exhibit a broader sensing range than unmodified samples while maintaining excellent piecewise linearity over 0–100 kPa. A high sensitivity of 0.157 kPa^−1^ is achieved at low pressure, with stable performance maintained at higher pressures. Cyclic and repeatability tests confirm their robustness and reliability.

Through combined process optimization, modeling, experiments, and simulations, this work mitigates particle sedimentation-induced non-uniformity in Cu/PDMS dielectrics. The resulting sensor delivers wide-range detection, high linearity, strong stability, and enhanced low-pressure sensitivity. This strategy improves particle dispersion in conductive composites and provides insight for designing flexible capacitive pressure sensors for wearable health monitoring and electronic skin.

## 2. Working Principle of the Capacitive Pressure Sensor

### 2.1. Structure of the Capacitive Pressure Sensor

**Figure 1:**
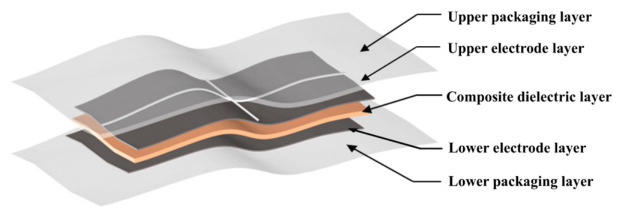
Capacitive sensor structure diagram.

As shown in Figure 1, the flexible capacitive pressure sensor features a five-layer symmetric structure comprising an upper encapsulation, an upper electrode array, a Cu/PDMS dielectric layer, a lower electrode, and a lower encapsulation. The device dimensions are 11 mm × 11 mm × 1 mm. Flexible silver-coated nylon conductive fabric (0.1 mm thick, sheet resistance < 1 Ω/sq) was used as the electrode material to ensure stable electrical conductivity and mechanical flexibility. The upper electrode is patterned into four 5 mm × 5 mm sensing units, while the lower electrode forms a continuous 11 mm × 11 mm layer to enhance capacitive coupling and minimize crosstalk. PDMS encapsulation provides structural protection while maintaining flexibility and durability. With the Cu/PDMS dielectric layer and compatible electrodes, the sensor is suitable for wearable electronics and robotic tactile sensing.

### 2.2. Working Principle Under Normal Pressure

**Figure 2:**
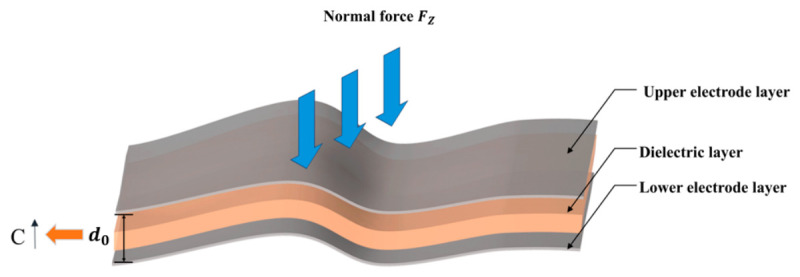
Schematic illustration of the applied normal force on the flexible capacitive pressure sensor.

Flexible capacitive pressure sensors operate based on the parallel-plate capacitor model. As shown in Figure 2, when an external normal pressure is applied, the dielectric layer undergoes elastic deformation, which reduces the distance between the electrodes. Consequently, the sensor capacitance increases with increasing applied pressure.

Neglecting edge effects, the initial capacitance $C_{0}$ of a parallel-plate capacitor can be expressed as follows:

(1) $$C_{0}=\frac{\varepsilon_{0}\varepsilon_{r}A}{d_{0}}$$

where $\varepsilon_{0}$ is the vacuum permittivity, $\varepsilon_{r}$ is the relative permittivity of the dielectric layer, A is the effective electrode area, and $d_{0}$ is the initial inter-electrode distance. When a normal pressure P is applied, the dielectric layer is compressed, and the electrode spacing decreases from $d_{0}$ to $d=d_{0}-\Delta d$, where Δd is the change in inter-electrode distance. Although a slight lateral expansion of PDMS under compression may lead to an increase in the effective electrode area *A*, its contribution to the overall capacitance variation is negligible compared with through-thickness deformation. Therefore, for simplicity of analysis, only the compressive strain along the thickness direction is considered. The capacitance under applied pressure can then be expressed as follows:

(2) $$C=\varepsilon_{0}\varepsilon_{r}\frac{A}{d}\;,\;\;d=d_{0}-\Delta d$$

Accordingly, the relative change in capacitance is expressed as follows:

(3) $$\frac{\Delta C}{C_{0}}=\frac{C-C_{0}}{C_{0}}=\frac{\Delta d}{d_{0}-\Delta d}$$

This relationship shows that, under the ideal condition of constant permittivity, the relative capacitance change is directly proportional to the relative change in inter-electrode distance. This forms the basic mechanism for converting pressure into an electrical signal in capacitive pressure sensors [14,41]. The deformation of the dielectric layer under applied pressure is described by Hooke’s generalized law:

(4) $$P=\sigma =E\varepsilon =E\cdot \frac{\Delta d}{d_{0}}$$

where σ is the stress, E is the elastic modulus of the dielectric layer, and $\varepsilon =\Delta d/d_{0}$ is the axial strain. The initial sensitivity S of the sensor is defined as the partial derivative of capacitance with respect to pressure [42]:

(5) $$\;S=\frac{\partial \left(\Delta C/C_{0}\right)}{\partial P}=\frac{\varepsilon_{0}\varepsilon_{r}A}{d_{0}E{\left(1-\frac{P}{E}\right)}^{2}}\;$$

When P≪E, that is, P/E→0, the above equation can be simplified as follows:

(6) $$S=\frac{\varepsilon_{0}\varepsilon_{r}A}{d_{0}}\cdot \frac{1}{E}=\frac{C_{0}}{E}$$

This relationship shows that the initial sensitivity of the sensor is directly proportional to the initial capacitance $C_{0}$ and inversely proportional to the elastic modulus E of the dielectric layer [43]. Therefore, increasing $C_{0}$ is a key approach for enhancing sensor sensitivity, while improving the relative permittivity $\varepsilon_{r}$ of the dielectric layer is a fundamental strategy to increase $C_{0}$ [26,44,45].

### 2.3. Theoretical Analysis of Non-Uniform Cu Particle Effects on Capacitance

**Figure 3:**
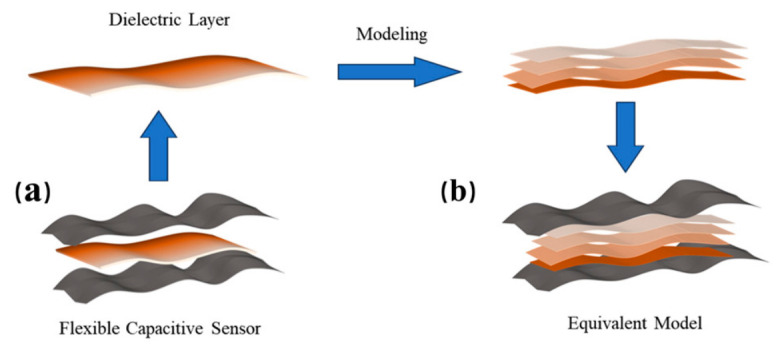
Establishment of the equivalent multilayer dielectric model for the flexible capacitive pressure sensor: (**a**) original sensor structure with a single dielectric layer; (**b**) simplified equivalent model for theoretical analysis.

To investigate the impact of sedimentation-induced non-uniform Cu particle distribution on sensor performance, a theoretical model was established by combining percolation theory with an equivalent series-capacitance approach. As illustrated in Figure 3, Cu particles are assumed to be linearly distributed along the thickness of the dielectric layer, with a higher concentration at the bottom and lower concentration at the top, while preserving the total filler content, expressed as $P_{\mathrm{avg}}=(P_{\max}+P_{\min})/2$. When the local Cu concentration is below the percolation threshold $P_{c}$, the relative permittivity can be described as $\varepsilon_{r}(P)=k(P_{c}-P{)}^{-t}$, where k is a proportionality coefficient and t is the critical exponent [46,47]. The dielectric layer can therefore be treated as a series of infinitesimal sub-capacitors along its thickness, and the overall capacitance is calculated using the series-capacitance model.

For both the uniform and non-uniform distribution models, the elastic modulus E, electrode area A, and total dielectric thickness $d_{0}$ were kept constant, so that only the effect of Cu particle distribution was considered. Assuming that z denotes the coordinate along the thickness of the dielectric layer, with z=0 at the bottom and $z=d_{0}$ at the top, the Cu concentration is described by a linear distribution:

(7) $$P(z)=P_{max}-\frac{P_{max}-P_{min}}{d_{0}}z$$

where $P_{max}$ and $P_{min}$ represent the maximum Cu concentration at the bottom and the minimum at the top, respectively. Substituting this concentration profile into the permittivity model gives the thickness-dependent relative permittivity:

(8) $$\varepsilon_{r}\left(z\right)=k{\left[P_{c}-P_{\max}+\frac{P_{\max}-P_{\min}}{d_{0}}z\right]}^{-t}$$

According to the equivalent series-capacitance model, the total capacitance of the flexible capacitor is given by the following [48]:

(9) $$\frac{1}{C}=\frac{1}{\varepsilon_{0}A}\int_{0}^{d_{0}}\frac{dz}{\varepsilon_{r}\left(z\right)}$$

After substituting $\varepsilon_{r}(z)$ into the above equation and performing the integration, the total capacitance under a non-uniform particle distribution can be expressed as follows:

(10) $$C_{\mathrm{non}-\mathrm{uni}}=\frac{\varepsilon_{0}Ak\left(t+1\right)\left(P_{\max}-P_{\min}\right)}{d_{0}\left[{\left(P_{c}-P_{\min}\right)}^{t+1}-{\left(P_{c}-P_{\max}\right)}^{t+1}\right]}$$

For the uniformly distributed case, the local Cu concentration equals the average concentration: $P_{\mathrm{avg}}=(P_{\max}+P_{\min})/2$. The relative permittivity is therefore constant, and the initial capacitance is given by the following:

(11) $$\begin{array}{c} C_{\mathrm{uni}}=\frac{\varepsilon_{0}A\varepsilon_{r,\mathrm{uni}}}{d_{0}}=\frac{\varepsilon_{0}Ak{\left(P_{c}-P_{\mathrm{avg}}\right)}^{-t}}{d_{0}} \end{array}$$

**Figure 4:**
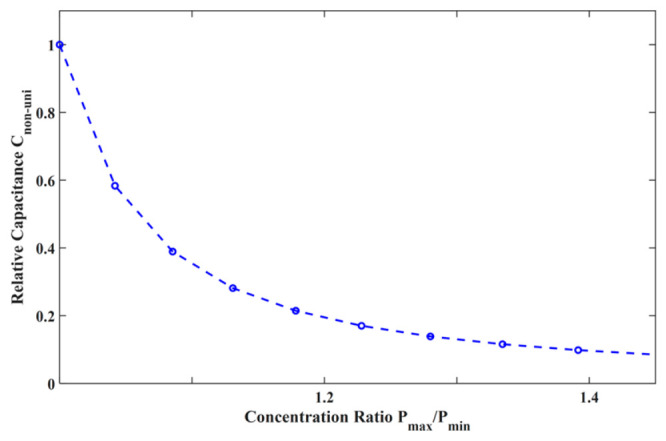
Relative capacitance $C_{\mathrm{non}-\mathrm{uni}}$ of the sensor as a function of the concentration ratio $P_{\max}/P_{\min}$.

Under constant total Cu content, the degree of particle distribution non-uniformity, defined by the concentration ratio $P_{\max}/P_{\min}$, significantly affects the equivalent capacitance of the composite dielectric layer. As shown in Figure 4, the normalized capacitance $C_{\mathrm{non}-\mathrm{uni}}$ decreases monotonically with increasing non-uniformity.

The mechanism can be summarized as follows. As the Cu particle distribution becomes more non-uniform, the Cu concentration in the upper region decreases, increasing the equivalent series-capacitance resistance and reducing $C_{non-uni}$. Although the high Cu concentration at the bottom locally enhances permittivity, it cannot compensate for the capacitance suppression caused by the low-concentration region. Consequently, particle sedimentation lowers the initial capacitance $C_{0}$ and, according to the sensitivity relationship, reduces sensor sensitivity. Therefore, uniform dispersion of Cu particles in the PDMS matrix is essential for improving effective permittivity, capacitance output, and the overall sensing performance of Cu/PDMS flexible capacitive pressure sensors.

## 3. Fabrication of the Flexible Pressure Sensor

### 3.1. Assembly of the Ultrasonic-Assisted Homogenization System

**Figure 5:**
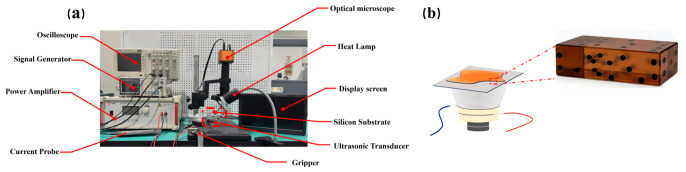
(**a**) Photograph of the ultrasonic-assisted homogenization system and its main components. (**b**) Schematic illustration of the ultrasonic-assisted dispersion mechanism of conductive fillers in the composite dielectric layer.

Ultrasonic system setup was shown in Figure 5, in which the silicon substrate was rigidly bonded to the vibrating surface of a Langevin ultrasonic transducer using acrylic AB adhesive to ensure efficient transmission of vibrational energy. The transducer was driven by a signal generator and a power amplifier, while an oscilloscope monitored the phase difference between voltage and current in real time to maintain resonance. A heating lamp was integrated into the system to maintain the temperature at approximately 50 °C, accelerating slurry curing. Simultaneously, an optical microscope enabled real-time observation to prevent excessive system gain from causing Cu particle agglomeration, thereby ensuring stable operation and experimental reliability.

### 3.2. Fabrication and Characterization of the Dielectric Layer

**Figure 6:**
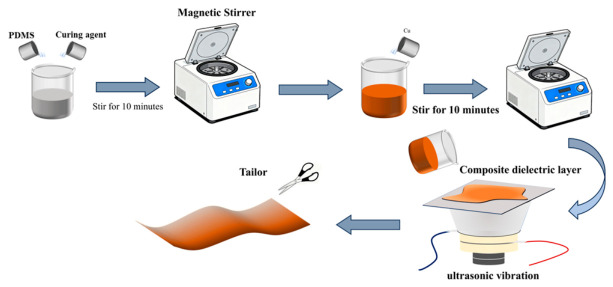
Schematic illustration of the fabrication process of the Cu/PDMS composite dielectric layer.

As illustrated in Figure 6, PDMS prepolymer and curing agent were weighed at a mass ratio of 10:1, added to a beaker, and mixed with a vacuum magnetic stirrer for 10 min. This ensured uniform mixing and removed trapped air bubbles. Pretreated Cu powder was added at the designed mass fraction and stirred for another 10 min to achieve initial dispersion in the PDMS matrix.

**Figure 7:**
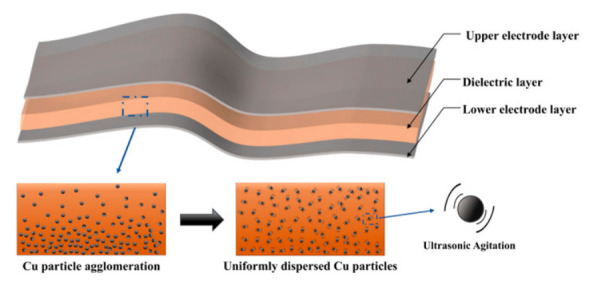
Ultrasonic-assisted homogenization of Cu particles in the PDMS matrix.

Slurries were prepared and cured using an ultrasonic-assisted homogenization system to promote uniform dispersion. The signal generator supplied a 1 V sinusoidal signal at 71.4 kHz to operate under resonance condition. The use of a higher-frequency acoustic field enables shorter wavelengths and a high density of pressure nodes, which facilitates efficient microparticle manipulation and redistribution within the PDMS matrix. The power amplifier gain was set to 15. The well-mixed Cu/PDMS slurry was cast onto a silicon substrate. Under ultrasonic vibration and gravity, the slurry self-leveled to a thickness of 1 mm. As shown in Figure 7, high-frequency ultrasonic vibration suppressed Cu particle sedimentation and agglomeration. This resulted in more uniform particle dispersion in the PDMS matrix. The sample was then cured at 50 °C for 2 h under continuous ultrasonic excitation to stabilize the uniform particle structure.

**Figure 8:**
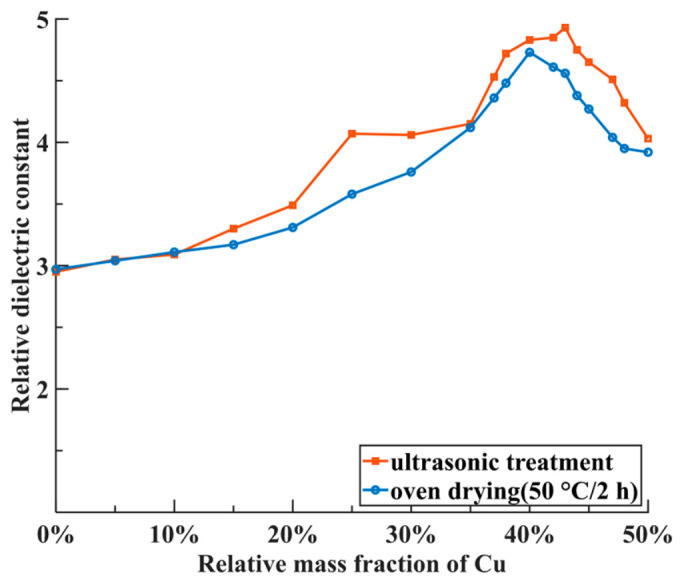
Variation in the relative dielectric constant of Cu/PDMS composites with Cu mass fraction for oven-dried and ultrasonic-treated samples.

The relative permittivity $\varepsilon_{r}$ of the flexible dielectric layer with different Cu mass fractions was measured using a high-precision LCR meter. Parallel-plate electrodes defined a fixed area. Measurements were conducted at 1 V and 100 kHz. Normal pressure was applied using a push–pull force gauge, and the corresponding capacitance was recorded five times at each pressure level. The average value was then calculated to improve reliability. The dielectric thickness was measured by optical microscopy with five repetitions. Finally, $\varepsilon_{r}$ was calculated from the measured capacitance and average thickness. The results are shown in Figure 8.

Figure 8 shows the relative permittivity of Cu/PDMS composites with different Cu contents prepared by different methods. In the ultrasonic group, the samples were treated as described above, while in the oven-dried group, the samples were dried at 50 °C for 2 h. Both groups display typical percolation behavior, with permittivity increasing gradually at low Cu concentrations, rising sharply near the critical region, and decreasing beyond the percolation threshold. At 0–20% Cu, the composites remain insulating, with permittivity around 3–3.5, showing little difference between the two methods. At 35–40% Cu, the shorter interparticle distance enhances interfacial polarization. The permittivity reaches ~4.7 for the oven-dried samples and ~4.95 for the ultrasonically treated samples. Beyond this range, continuous conductive pathways form, and leakage current lowers the permittivity. Ultrasonic treatment improves Cu dispersion and reduces agglomeration, resulting in higher permittivity at medium- and high-concentration regions. Therefore, ~40% Cu is selected as the optimal composition for high-sensitivity flexible capacitive pressure sensors.

### 3.3. Effect of Ultrasonic Excitation Modes on Permittivity

To study the effect of driving signals on the dielectric properties of Cu/PDMS composites, sinusoidal and square waves were used to drive the ultrasonic transducer. Based on preliminary tests, an amplification factor of 15 was selected to avoid Cu agglomeration and ensure consistent experimental conditions.

Since percolation in Cu/PDMS occurs at high concentrations, Cu mass fractions of 35–48% were selected to examine the percolation region. The effects of the two driving signals on peak permittivity and percolation behavior were then compared. Key processing parameters, including ultrasonic frequency and curing temperature/time, were kept constant. Samples with different Cu concentrations were prepared and tested using the same protocol.

Dielectric performance was evaluated by plotting relative permittivity against Cu concentration. This enabled a direct comparison of the two excitation modes and provided a basis for optimizing the ultrasonic driving signal.

**Figure 9:**
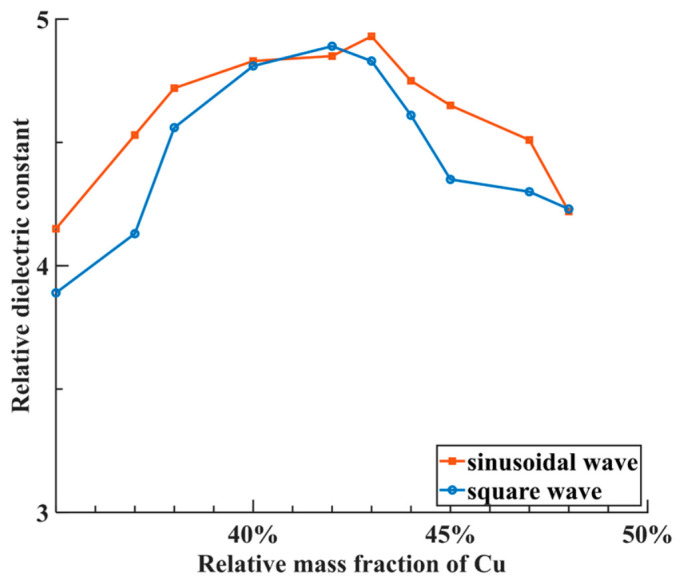
Variation in the relative dielectric constant of Cu/PDMS composites with Cu mass fraction under different ultrasonic waveform treatments.

As illustrated in Figure 9, the peak permittivity values of the two excitation modes are similar. The sinusoidal-wave-driven sample shows a slightly higher value (~4.9) than the square-wave-driven sample (~4.8). The difference is more evident at low Cu concentrations (35–40%). At higher concentrations, the sinusoidal-driven sample shows a slower decrease. This can be attributed to the more uniform energy distribution of the sinusoidal ultrasonic field, which improves Cu dispersion in the PDMS matrix, reduces agglomeration, and delays conductive pathway formation. As a result, strong interfacial polarization is maintained over a wider concentration range. Overall, sinusoidal excitation is a more effective ultrasonic strategy for fabricating high-sensitivity flexible pressure sensors. Therefore, the following assembly process employed dielectric layers with Cu content around 40%, prepared under sinusoidal wave driving.

### 3.4. Assembly of the Flexible Pressure Sensor

**Figure 10:**
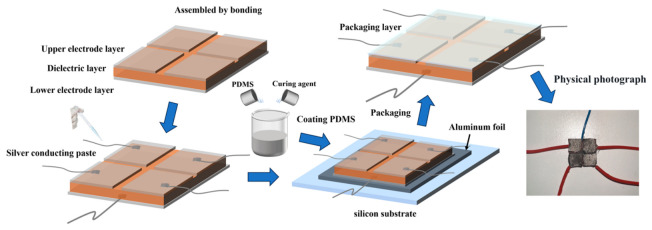
Fabrication process of the flexible capacitive pressure sensor array.

As illustrated in Figure 10, conductive fabric was used as the electrode material. The upper electrode was patterned into four 5 mm × 5 mm array units, while the lower electrode formed a continuous 11 mm × 11 mm layer to reduce signal interference between adjacent units. The sensor employed a five-layer structure consisting of an upper encapsulation layer, an upper electrode array, a Cu/PDMS composite dielectric layer, a lower electrode, and a lower encapsulation layer. Electrical leads were bonded to the electrodes with conductive silver paste and cured at room temperature. Finally, PDMS was coated on both surfaces and cured to complete encapsulation, forming an integrated flexible capacitive pressure sensor.

## 4. Performance Evaluation of the Flexible Pressure Sensor

### 4.1. Performance Evaluation of an Individual Sensing Unit

Quasi-static compression tests were performed to evaluate the normal-force response of the flexible capacitive sensor. A stepper-motor-driven linear stage and a digital push–pull force gauge were used to apply and monitor normal pressure in the range of 0 to 100 kPa. To minimize capacitance fluctuations during LCR measurements caused by load impact, both loading and unloading were performed at a slow rate of 4 cm/s. After each target displacement was reached, the load was maintained for 2 s to stabilize the capacitance signal before unloading at the same rate. Each measurement point was repeated five times, and the averaged values were used to reduce random errors and improve data reliability.

**Figure 11:**
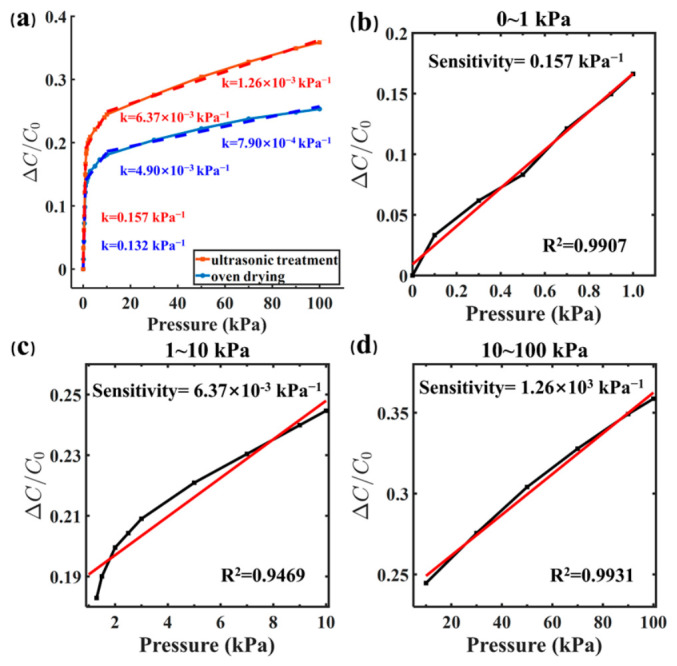
Relative capacitance change as a function of external pressure in different pressure ranges: (**a**) 0–100 kPa; (**b**) 0–1 kPa; (**c**) 1–10 kPa; (**d**) 10–100 kPa.

Capacitance responses under different normal pressures were measured using an LCR meter. The sensing performance was evaluated from the relative capacitance change $\Delta C/C_{0}$. The sensitivity was calculated according to $S=\frac{\Delta C/C_{0}}{\Delta P}$, where ΔP represents the applied pressure change. As presented in Figure 11, both Cu/PDMS flexible capacitive sensors showed a typical piecewise-linear pressure response. The sensitivity was highest in the low-pressure range of 0–1 kPa, reaching 0.157 kPa^−1^ for the ultrasonically treated sample and 0.132 kPa^−1^ for the oven-dried sample. The ultrasonically treated sample also showed good linearity, with $R^{2}=0.9907$. As pressure increased, the sensitivity decreased to 0.00637 and 0.00490 kPa^−1^ in the 1–10 kPa range, and further to 1.26 and 0.79 MPa^−1^ in the 10–100 kPa range for the ultrasonically treated and oven-dried samples, respectively. The ultrasonically treated sensor still maintained excellent linearity in the high-pressure region, with $R^{2}=0.9931$.

Comparative analysis shows that ultrasonic treatment gives the sensor higher sensitivity and better linearity over the full pressure range, especially at high pressure. This improvement is mainly due to better Cu particle dispersion in the PDMS matrix, which reduces agglomeration and forms a more uniform microcapacitive network. As a result, the permittivity changes more effectively under pressure, improving the force-sensing performance. The high sensitivity at low pressure also indicates good potential for weak-force detection.

**Figure 12:**
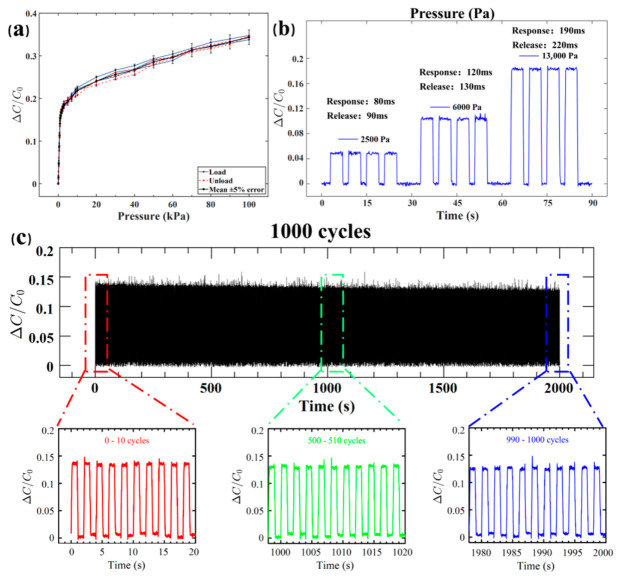
Performance characterization of the flexible capacitive pressure sensor: (**a**) loading–unloading curves of relative capacitance change $\Delta C/C_{0}$ versus pressure; (**b**) repeatability test under different applied pressures (2500 Pa, 6000 Pa, and 13,000 Pa); (**c**) durability test over 1000 loading cycles, with enlarged views of the initial, middle, and final 10 cycles.

As shown in Figure 12a, the sensor response was evaluated under repeated loading–unloading cycles. The error bars indicate a measurement uncertainty of ±5%. The loading curve (solid blue line) and unloading curve (red dashed line) largely overlap, with only small deviations in limited regions. This result indicates consistent loading and unloading behavior, demonstrating good repeatability and low hysteresis. Therefore, the sensor provides a stable relationship between applied pressure and capacitance change.

Figure 12b shows the repeatability of the sensor under different pressure levels, including 2500 Pa, 6000 Pa, and 13,000 Pa. The relative capacitance increases steadily with applied pressure, and the stable capacitance response confirms the reliability of the sensor output.

Flexible pressure sensors require good mechanical durability to maintain stable input–output behavior under long-term or cyclic loading conditions. Therefore, cyclic loading tests were conducted at 0.8 kPa and 0.5 Hz. As shown in Figure 12c, the sensor was tested over 1000 loading cycles. Three representative stages were selected for analysis, and their waveforms remained highly consistent. Although the capacitance change rate decreased slightly with increasing cycle number, the standard deviation over all cycles remained within 6%. These results confirm the excellent long-term stability and reliability of the sensor.

### 4.2. Performance Evaluation of the Sensor Array

**Figure 13:**
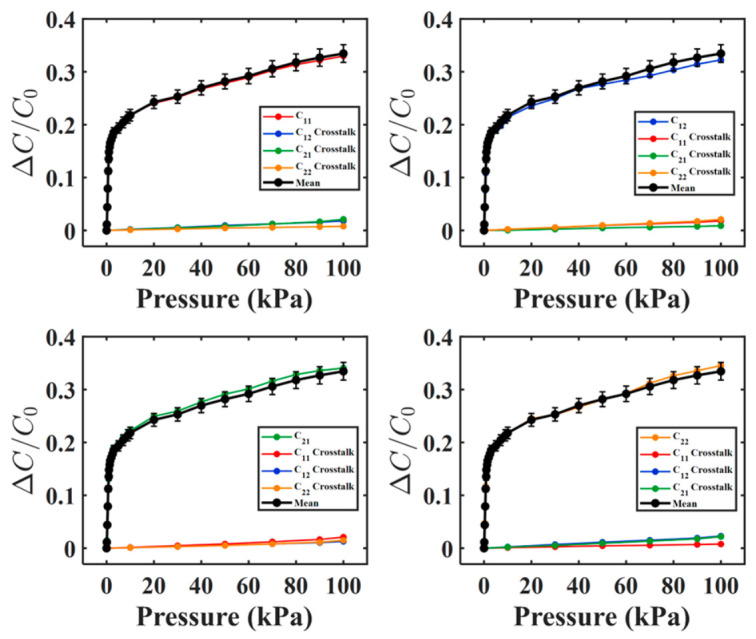
Pressure response curves of the four sensing units ($C_{11}$, $C_{12}$, $C_{21}$, and $C_{22}$) in the flexible capacitive pressure sensor array, showing the effects of crosstalk between adjacent units.

As shown in Figure 13, the pressure responses of four units ($C_{11}$, $C_{12}$, $C_{21}$, and $C_{22}$) in the array-structured flexible sensor were measured over the pressure range of 0–100 kPa. The results show good consistency among the sensing units and stable overall performance.

## 5. Finite Element Simulation and Dielectric Analysis of the Sensor

Finite element simulations were performed to investigate the effects of Cu particle distribution and concentration on the sensor’s dielectric performance. The electric potential, electric field, and current density were analyzed under various conditions. These results provide theoretical guidance for optimizing the Cu/PDMS dielectric layer and enhancing the performance of flexible capacitive pressure sensors.

### 5.1. Three-Dimensional Simulation of the Cu/PDMS Dielectric Layer

To capture the effect of ultrasonic-assisted dispersion on the microstructure of the composite dielectric, two representative particle distribution configurations were considered: a uniformly dispersed configuration and a sedimented configuration. A three-dimensional current-field simulation model was developed to investigate the effects of Cu particle distribution and concentration on the capacitance, effective permittivity, and internal electric-field distribution of the dielectric layer. In the simulation domain, the Cu/PDMS dielectric layer was assigned the corresponding electrical properties. Specifically, Cu particles were assigned an electrical conductivity of $5.8\times 10^{7}\;S/m$ and a relative permittivity of 1, while PDMS was assigned a conductivity of $1\times 10^{-12}\;S/m$ and a relative permittivity of 2.78. The upper electrode was set to $V_{1}=1\;V$, whereas the lower electrode was grounded $\left(V_{2}=0\;V\right)$. All outer boundaries were treated as electrically insulating. A sinusoidal voltage at 50 Hz was applied under frequency-domain analysis.

The system was treated as a complex impedance network, and the capacitive response was characterized by the complex admittance. Inductive effects were neglected for the dielectric structure. Under sinusoidal steady-state conditions, the complex admittance of an ideal capacitor is expressed as follows [49]:

(12) Y=jωC

where ω=2πf is the angular frequency, f is the AC excitation frequency, and C is the equivalent capacitance. In the simulation, the frequency-domain current-field equations were solved using the finite element method to obtain the complex admittance between the electrodes. The capacitance was then calculated from the imaginary part of the admittance:

(13) C=ImYω

**Figure 14:**
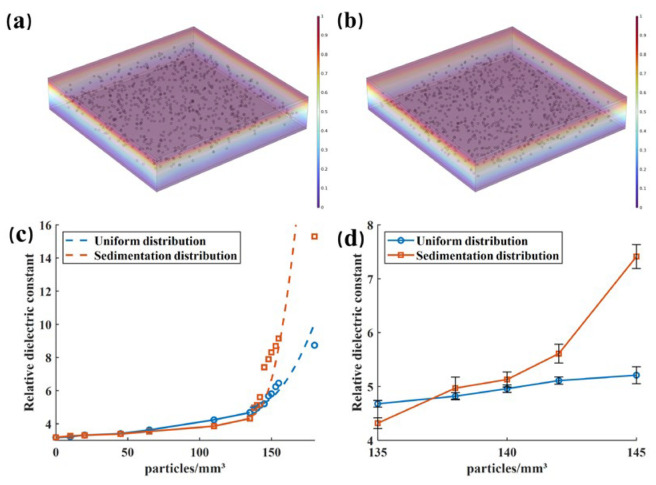
(**a**,**b**) Three-dimensional potential distributions of Cu/PDMS composites with uniform and sedimented Cu particle distributions. (**c**,**d**) Relative permittivity as a function of Cu concentration, including overall and enlarged views.

The equivalent capacitance was obtained from the imaginary part of the complex admittance, and the relative permittivity was calculated using the governing capacitance equation. As shown in Figure 14, uniform and sedimented Cu/PDMS dielectric models were constructed to evaluate the effect of Cu particle concentration on permittivity. The results show that relative permittivity increases with Cu concentration in both models, with clear differences emerging at high concentrations. In the sedimented model, permittivity rises sharply at 130–150 mm^−3^, indicating percolation behavior near 140 mm^−3^. The abnormal increase at higher concentrations is due to the absence of PDMS breakdown effects in the simulation. In contrast, the uniform model shows a more gradual increase, suggesting better dielectric stability.

To correlate the simulated particle concentration with the experimentally used Cu loading, the corresponding mass fraction was estimated. Assuming spherical Cu particles, the particle volume is expressed as $V_{p}=\frac{4}{3}\pi r^{3}$, where r is the particle radius. Since the particle concentration in the simulation is defined as the number of particles per unit volume, the Cu mass fraction relative to PDMS can be calculated by the following:

(14) $$C_{wt}=\frac{N\left(\frac{4}{3}\pi r^{3}\right)\rho_{Cu}}{\left(1-N\frac{4}{3}\pi r^{3}\right)\rho_{PDMS}}\times 100\%$$

where N is the particle number density ($mm^{-3}$), and $\rho_{Cu}$ and $\rho_{PDMS}$ are the densities of Cu and PDMS, respectively.

### 5.2. Two-Dimensional Electric-Field Analysis of the Dielectric Layer

To improve computational accuracy and efficiency, we simplified the capacitive structure into a two-dimensional planar model to study electric potential, electric field, current density, and capacitance. Preliminary simulations showed that the uniform model had clear percolation behavior at 40–50 particles mm^−3^. Therefore, we selected three representative concentrations near the threshold, 40, 45, and 50 mm^−3^, for detailed field analysis. All heat maps of the same quantity used the same color scale for direct comparison under different conditions.

**Figure 15:**
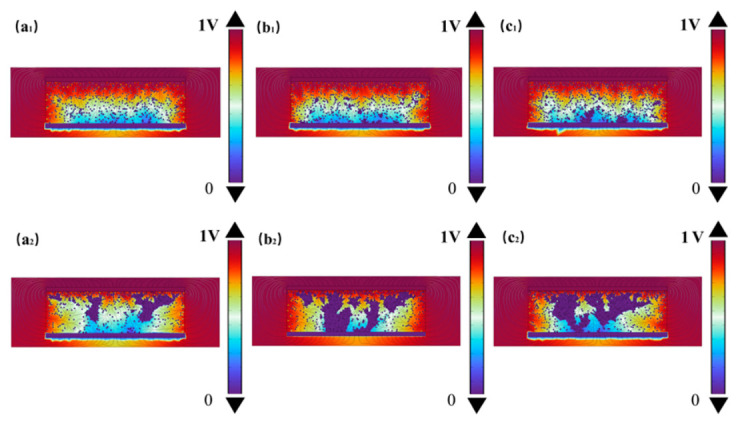
Electric potential distribution in Cu/PDMS composite dielectric layers with different Cu particle concentrations (40, 45, and 50 particles/mm^2^, corresponding to (**a**), (**b**), and (**c**)) under two dispersion states. The upper row (**a_1_**–**c_1_**) shows the potential field in uniformly dispersed samples, while the lower row (**a_2_**–**c_2_**) shows the potential field in sedimented samples with particle agglomeration.

As shown in Figure 15, the electric potential near the upper electrode in the sedimented model drops sharply. This is mainly due to the accumulation of Cu particles in this region. At the same time, multiple purple island-like equipotential domains are formed within the dielectric layer. Within these islands, Cu particles form locally connected networks that act as equipotential bodies. As a result, the internal potential quickly equilibrates, and the potential difference within each island becomes negligible. Pronounced potential gradients appear at the interfaces between these islands and the surrounding dielectric matrix. These gradients can distort the local electric field and deviate from the ideal uniform field of a parallel-plate capacitor.

**Figure 16:**
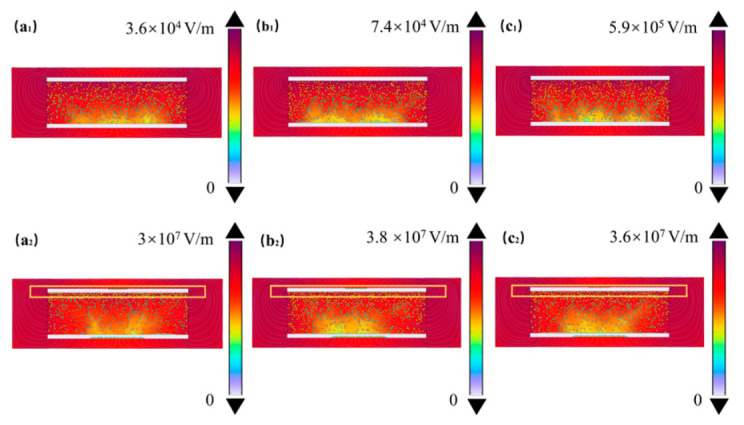
Electric-field intensity distribution in Cu/PDMS composite dielectric layers with different Cu particle concentrations (40, 45, and 50 particles/mm^2^, corresponding to (**a**), (**b**), and (**c**)) under two dispersion states. The upper row (**a_1_**–**c_1_**) shows the electric field in uniformly dispersed samples, while the lower row (**a_2_**–**c_2_**) shows the electric field in sedimented samples with particle agglomeration.

As shown in Figure 16, the electric-field distribution exhibits distortion and concentration within the composite dielectric. Cu particles act as equipotential bodies, leading to strong field enhancement at particle edges, especially near the electrodes and in narrow interparticle gaps. These regions are potential sites for local dielectric breakdown. Averaged simulation results indicate that the maximum electric field in the sedimented model reaches $3.8\times 10^{7}V/m$ at 45 mm^−3^, which exceeds the typical dielectric breakdown strength of PDMS, generally in the range of $2.5–3.0\times 10^{7}V/m$. The high field is mainly concentrated near Cu particle–electrode contact regions and can exceed the PDMS breakdown strength under certain conditions. In contrast, the uniform model at the same concentration shows a much lower maximum field of $7.4\times 10^{4}V/m$, indicating a higher insulation safety margin.

**Figure 17:**
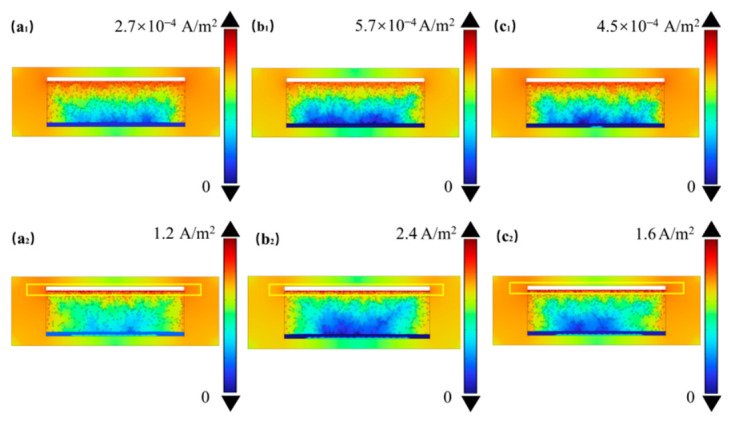
Current-density distribution in Cu/PDMS composite dielectric layers with different Cu particle concentrations (40, 45, and 50 particles/mm^2^, corresponding to (**a**), (**b**), and (**c**)) under two dispersion states. The upper row (**a_1_**–**c_1_**) shows the current density in uniformly dispersed samples, while the lower row (**a_2_**–**c_2_**) shows the current density in sedimented samples with particle agglomeration.

As shown in Figure 17, the current-density distribution reveals internal conduction pathways and potential leakage channels within the composite dielectric. Due to the high conductivity of Cu particles, current concentrates mainly within the particles. Pronounced current-density peaks also appear in the PDMS gaps between particles and at particle edges near the electrodes because of local electric-field distortion. These regions may form pre-breakdown channels, which can develop into continuous conductive paths under increased voltage, leading to dielectric failure. Averaged simulation results show that the maximum current density in the sedimented model reaches $2.4\;A/m^{2}$ at 45 ${mm}^{-3}$, whereas the uniform model at the same concentration shows a maximum current density of only $5.7\times 10^{-4}\;A/m^{2}$. This result indicates that uniform particle distribution provides a much higher breakdown safety margin, even at high filler concentrations.

Comparative current-field simulations indicate that Cu particle distribution strongly affects the electric potential, electric field, and current-density behavior of the dielectric layer. Particle sedimentation, compared with the uniform model, induces local field concentration and current crowding. This lowers breakdown tolerance and increases the risk of insulation failure. In contrast, uniform Cu dispersion suppresses field distortion and leakage pathways, improving the dielectric reliability and operational stability of Cu/PDMS flexible capacitive pressure sensors.

## 6. Conclusions

In this work, a flexible capacitive pressure sensor with a Cu/PDMS composite dielectric layer was fabricated using ultrasonic-assisted homogenization. Ultrasonic vibration was applied to suppress Cu particle sedimentation and agglomeration, resulting in uniform particle dispersion in the PDMS matrix. Based on percolation theory and an equivalent series-capacitance model, the relationship between particle distribution and dielectric properties was established. Finite element simulations were conducted to analyze the electric potential, electric field, and current-density distributions under different particle distributions. The results show that uniform particle dispersion reduces local electric-field concentration and current leakage, thereby improving dielectric stability and breakdown safety margin.

Experimental results show that ultrasonic treatment significantly improves the dielectric and pressure-sensing performance of the sensor. The sensor achieved a sensitivity of 0.157 kPa^−1^ in the low-pressure range, while the high-pressure sensitivity increased by approximately 60% compared with the untreated sample. The sensor also exhibited good piecewise linearity, repeatability, and cyclic stability over 0–100 kPa. Stable output was maintained after 1000 loading cycles, indicating good long-term reliability.

Overall, ultrasonic-assisted homogenization demonstrates potential as a useful method for regulating conductive particle dispersion in composite dielectric layers, thereby contributing to the dielectric and sensing performance of flexible capacitive pressure sensors. The proposed approach may offer insights for the design and development of sensors in applications including human–machine interaction, wearable health monitoring, and electronic skin.

## Data Availability

The original contributions presented in this study are included in the article. Further inquiries can be directed to the corresponding author.
